# On Treatment of Spinal Curvature by Reclination in Its Early Stages

**Published:** 1883-11

**Authors:** Edward Lund

**Affiliations:** Professor of Surgery in the Owen’s College, Victoria University, Manchester


ON THE TREATMENT OF SPINAL CURVATURE BY RECLINATION IN ITS EARLY STAGES. Mr. Edward Lund, F.R.C.S.. Professor of Surgery in the Owen's College, Victoria University, Manchester, writes:

"I hope to exhibit, at the forthcoming meeting of the British Medical Association, at Liverpool, a form of couch for the treatment, by reclination, of spinal curvature in its early stage, and weakness of the muscles of the spine, which embodies in its action a principle of treatment for such cases too frequently overlooked.

"The couch which I have to recommend, and which will be shown at Liverpool, is designed to carry out by reclination the same principle of treatment as operates in the method of vertical suspension, only in a more gradual and prolonged manner. I

have called my couch a 'slippery couch,' and I think the construction and mode of action will justify the term. I have used it with marked benefit during the last few years, in more than thirty cases, in private practice. It is made in this way: A piece of wood is prepared, of suitable thickness, and about six feet long and eighteen inches wide. At about four inches from one end a hole is cut through the wood, of circular form and six inches in diameter, with its margin on one surface of the wood slightly beveled inwards. This end of the piece of wood is to be the upper or higher part, when it is fixed at such an inclination by means of a block or cross-piece as to raise it about one foot at the higher end. It is well to have four wooden legs screwed on, one at each corner, the upper pair being longer than the lower in the same proportion; and to still further influence the angle at which the couch is to be used, by means of extra screw holes in the wood; the longer pair of legs being brought nearer to the foot of the couch, a greater elevation can be secured. The flat piece of wood being so prepared, is covered with several folds of soft thick blanket to about two inches in thickness, the blanket being just the size of the wood on one surface only; over this a piece of well polished black horse-hair cloth is stretched, and being turned tightly over the edges of the board, is nailed underneath, so as to produce a smooth, somewhat soft, but yet slippery, almost polished surface. Where the blanket crosses over the hole already described, it must be cut across in two directions, longitudinally and transversely, and the horse-hair cloth should be left loose over the same spot, so that, if pressure be here applied, an indentation will be quickly made.

"Now, if a couch be prepared in this way, and placed at such an angle of elevation as I have here described, about one part in six of its length, a person lying upon it on his back will soon find, unless he makes some effort to resist, that he will quietly slide down towards the lower end of the couch; and if his attention is otherwise absorbed, he will have his feet over the end of the board, as he is sliding beyond it. By a very simple device, this tendency to slide or slip downwards may be very beneficially utilized for the object we have in view.

"A small, firm, cylindrical pillow is prepared, about the diameter of the wrist, and a foot in length, and this is attached by strong tapes, one at each end of the pillow, and fixed to each upper corner of the couch, the length of the tapes being such as to place the pillow tranversely on the board immediately below the lower edge of the hole in the wood. With this pillow in position, and the patient so placed that the pillow may be received into the recess of the nape of the neck, the projection of the occiput falling into the depression made by the hole in the wood, the body is retained in position, and the sliding down is prevented, but yet there is a constant gentle dragging action on the spinal joints from the weight of the pelvis and lower limbs, which will act most favorably in the required direction.

" It is desirable, when a patient uses this couch for the first time, that he should try it without the pillow; and, if needful, the elevation of the couch should be adjusted until the pecular sliding movement is experienced. Then, with the help of the pillow, and the back of the head falling into the recess prepared for it, the patient will be aware of the principle upon which the couch is intended to act, and be more likely to continue its use.

"All other couches, such as the Ilkley couch, and couches with a double angular bed to support the knees, or with a foot-piece against which the feet can rest, are entirely opposed in principle to the plan of this 'slippery couch.' Using them, the patient may feel rested, and experience some temporary relief; but I know of no way, by reclination, to secure a certain degree of spinal extension, better than to fix the upper segment of the vertebro-cranial axis at one spot, and allow the weight of the lower part to induce direct 'self-extension.' "—*British Medical Journal.*

